# Assessing the susceptibility of 200 *Rhipicephalus microplus* populations to fluralaner using larval immersion test: A nationwide analysis from Brazil

**DOI:** 10.1186/s13071-026-07364-7

**Published:** 2026-04-16

**Authors:** Haile Dean Figueiredo Chagas, Arthur Matos de Santana, Gabriel Webert Gomes, Ana Carolinne Lopes Ascenção, Emanuel Magalhães Souza, Laura Cristina Ferreira Faria, Mayara Macedo Barrozo, Ana Lucia Coutinho Teixeira, Daniel de Castro Rodrigues, Fernando de Almeida Borges, Lívio Martins Costa-Junior, Lorena Lopes Ferreira, Márcia Cristina de Azevedo Prata, Welber Daniel Zanetti Lopes, Caio Monteiro

**Affiliations:** 1 Graduate Program in Animal Science—School of Veterinary and Animal Science, Federal University of Goiás, Goiânia-Nova Veneza Highway, km 8, Campus Samambaia, Goiânia, GO CEP 74690-900 Brazil; 2 Undergraduate Program in Veterinary Medicine, Federal University of Goiás, Nova Veneza, km 8, Campus Samambaia, Goiânia, GO CEP 74690-900 Brazil; 3 Undergraduate Program in Biotechnology, Federal University of Goiás, R. 235, s/no—University East Sector, Goiânia, GO CEP 74605-050 Brazil; 4 MSD Animal Health, Avenida Dr. Chucri Zaidan, 246-96, 9th Floor, São Paulo, SP CEP 04583-110 Brazil; 5 Federal University of Mato Grosso do Sul, Av. Senador Felinto Muller, 2443, Campo Grande, MS 79070‑900 Brazil; 6 CCBS Research Center, Federal University of Maranhão, Avenida dos Santos, Portugueses, No. 1966, São Luís, MA 65080‑805 Brazil; 7 Department of Preventive Veterinary Medicine, School of Veterinary Medicine, Federal University of Minas Gerais, Belo Horizonte, MG CEP 31270-901 Brazil; 8 Parasitology Laboratory at Embrapa Dairy Cattle, R. Eugênio do Nascimento, 610, Dom Bosco, Juiz de Fora, MG CEP 36038-330 Brazil; 9 Department of Biosciences and Technology, Institute of Tropical Pathology and Public Health, Federal University of Goiás, R. 235, s/no, University East Sector, Goiânia, GO CEP 74605-050 Brazil

**Keywords:** Cattle tick, Discriminating dose, Isoxazoline, Larval immersion test, Field trial

## Abstract

**Background:**

In 2022, a tick control product, against *Rhipicephalus microplus*, containing fluralaner as the active ingredient was initially launched in the Brazilian market, followed by other markets across Latin America. Once a new molecule is introduced for the control of a parasitic organism, it becomes essential to develop methods for assessing the susceptibility of target parasitic populations. In this context, discriminating dose (DD) tests represent a valuable tool.

**Methods:**

This study evaluated the susceptibility profile of 200 *R. microplus* populations originating from 21 federal units (20 states and a federal district) across all five regions of Brazil, using a larval immersion test (LIT) with two discriminating doses (DD) of 1.55 and 3.16 µg/mL. Populations showing mortality rates above 95% were classified as susceptible. A field trial was also conducted with one population (São José do Rio Pardo [SJRP]), which had a known history of exposure to fluralaner, to compare laboratory and field test outcomes.

**Results:**

The DD at a concentration of 1.55 µg/mL resulted in 100% mortality in 160 populations (80%), whereas the DD at a concentration of 3.16 µg/mL showed more consistent results, with 100% mortality in 182 populations (91%). In the field trial population, SJRP, fluralaner achieved 100% therapeutic efficacy (days +7 to +21) and persistent efficacy (days +28 to +42).

**Conclusions:**

LIT using DDs can provide indicative data on the susceptibility of *R. microplus* to fluralaner. The discriminating dose at a concentration of 3.16 µg/mL was shown to be the most appropriate for monitoring the susceptibility of *R. microplus* populations to fluralaner. Laboratory and field data support the classification of the SJRP population as susceptible, demonstrating consistency between the two sets of results. These results can serve as a basis for continuous spatial and temporal monitoring of the susceptibility of *R. microplus* populations to fluralaner. Continued research, integrating laboratory and field results, is essential to increase the reliability of laboratory-based testing.

**Graphical Abstract:**

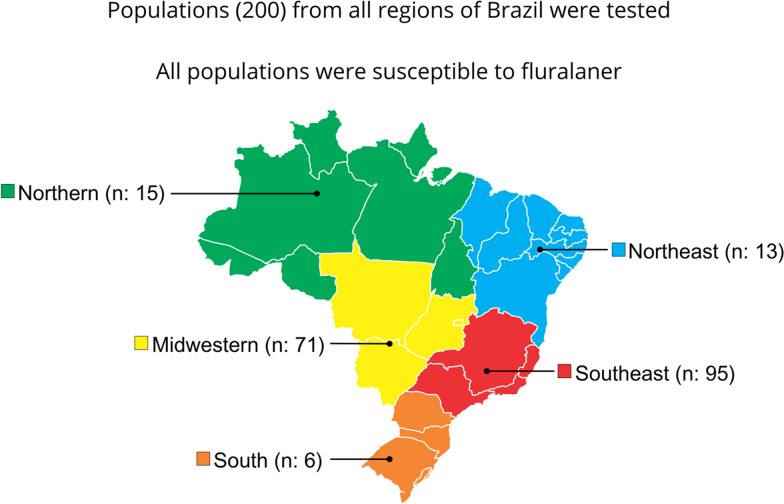

## Background

The cattle tick *Rhipicephalus microplus* (Canestrini, 1888; Acari: Ixodidae) is a hematophagous ectoparasite widely distributed across tropical and subtropical climate regions, included countries in the American, African, and Asian continents, causing significant economic losses in cattle farming in affected countries [1–3]. Brazil currently has the largest commercial cattle herd in the world, being the second-largest producer of beef and the fourth-largest producer of milk [4–6]. The presence of this tick leads to considerable production losses. In Brazil alone, the economic damage is estimated at around 3.2 billion USD annually [7].

Its control is almost exclusively carried out using chemical acaricides [2, 8–10]. However, the overuse and sometimes improper use of these chemicals has led to the development of resistance in many tick populations to almost all classes of acaricides available on the market [2, 9, 11]. In response to the growing resistance issue, there has been an urgent demand for the development of new control technologies, as no new class of acaricide had been introduced for over 25 years [1, 2, 12]. In 2022, a new drug class for the control of ectoparasites in cattle, including *R. microplus*, was initially launched in the Brazilian market and later introduced in other Latin American markets. The active ingredient of this product is fluralaner, a molecule from the isoxazoline class [13, 14].

The introduction of this new drug for the control of *R. microplus* highlights the importance of developing and validating methodologies to assess the susceptibility profiles of different populations. This would enable the implementation of management and control measures that can delay the emergence of resistant populations, thereby extending the efficacy of this molecule [1, 9, 15, 16]. The susceptibility profile of *R. microplus* populations to acaricides can be assessed through laboratory or field trials [10, 14, 16–20]. Among the available in vitro methodologies, although the use of discriminating doses (DDs) has some limitations, such as not providing information on dose–response curves, slope, or lethal concentration estimates (e.g., median lethal concentration causing death in 50% of the population [LC50]), it is worth noting that DDs offer important advantages. Specifically, DD-based assays require a smaller number of tested concentrations, and consequently fewer ticks, which allows the assessment of susceptibility in a larger number of populations [1, 14, 16, 20]. These characteristics are fundamental for enabling studies involving hundreds of samples, making this approach the best choice for this type of research [21, 22]. But the laboratory results should be interpreted as indicative, whereas field trials, though more challenging to conduct, should be considered conclusive. This is because studies comparing laboratory and field efficacy results have sometimes shown inconsistent findings [19, 20, 23–25].

For fluralaner, laboratory assays using the larval immersion test (LIT) [14] and the larval packet test (LPT) [19], providing information on dose–response curves, slope, and lethal concentration estimates, have already been described in the literature. Recently, two discriminating doses (DDs) for fluralaner were proposed using LIT. The first DD (1.55 µg/mL) was based on 2 × lethal concentration causing death in 99% of the population (LC99) of an acaricide-susceptible *R. microplus* strain [14], following established guidelines [1, 16, 26]. However, a recent study reported discrepancies between DD-based laboratory bioassays (based on 2 × LC99 of a susceptible strain) and field trial outcomes regarding the susceptibility of *R. microplus* to acaricides [19, 20], suggesting the need for new approaches to this type of research. Thus, Rodrigues et al. [14] also proposed a second DD (3.16 µg/mL) with fluralaner, based on 2 × LC99 calculated from pooled data of 18 populations with no prior exposure to fluralaner [14]. This proposal aimed at establishing a DD that is more representative of the tick species *R. microplus* since there is natural variability among populations.

In this type of investigation, to assess the reliability of these methodologies, it is important that laboratory results obtained using DDs are validated through continuous comparisons with field trials [14, 20]. More studies to investigate the relationship between bio-assay results and field efficacy are required to improve treatment recommendations and acaricide resistance management [5]. Thus, the objective of this study was to evaluate the phenotypic susceptibility profile of *R. microplus* populations from different regions of Brazil, using both the LIT/DD bioassays and a field trial.

## Methods

### Ticks

For the DD tests, 200 *R. microplus* populations were used. Being two reference strains (POA—a strain originated from the municipality of Porto Alegre, Rio Grande do Sul) and GYN (a strain originated from the municipality of Goiânia, Goiás) and samples from 198 *R. microplus* populations collected from different regions of Brazil. The POA strain is susceptible to all commercially available acaricide classes, whereas the GYN strain exhibits resistance to synthetic pyrethroids, formamidines, organophosphates, and phenylpyrazoles [25]. Ticks from the POA and GYN reference strains were maintained through artificial infestations in cattle.

**Fig. 1:**
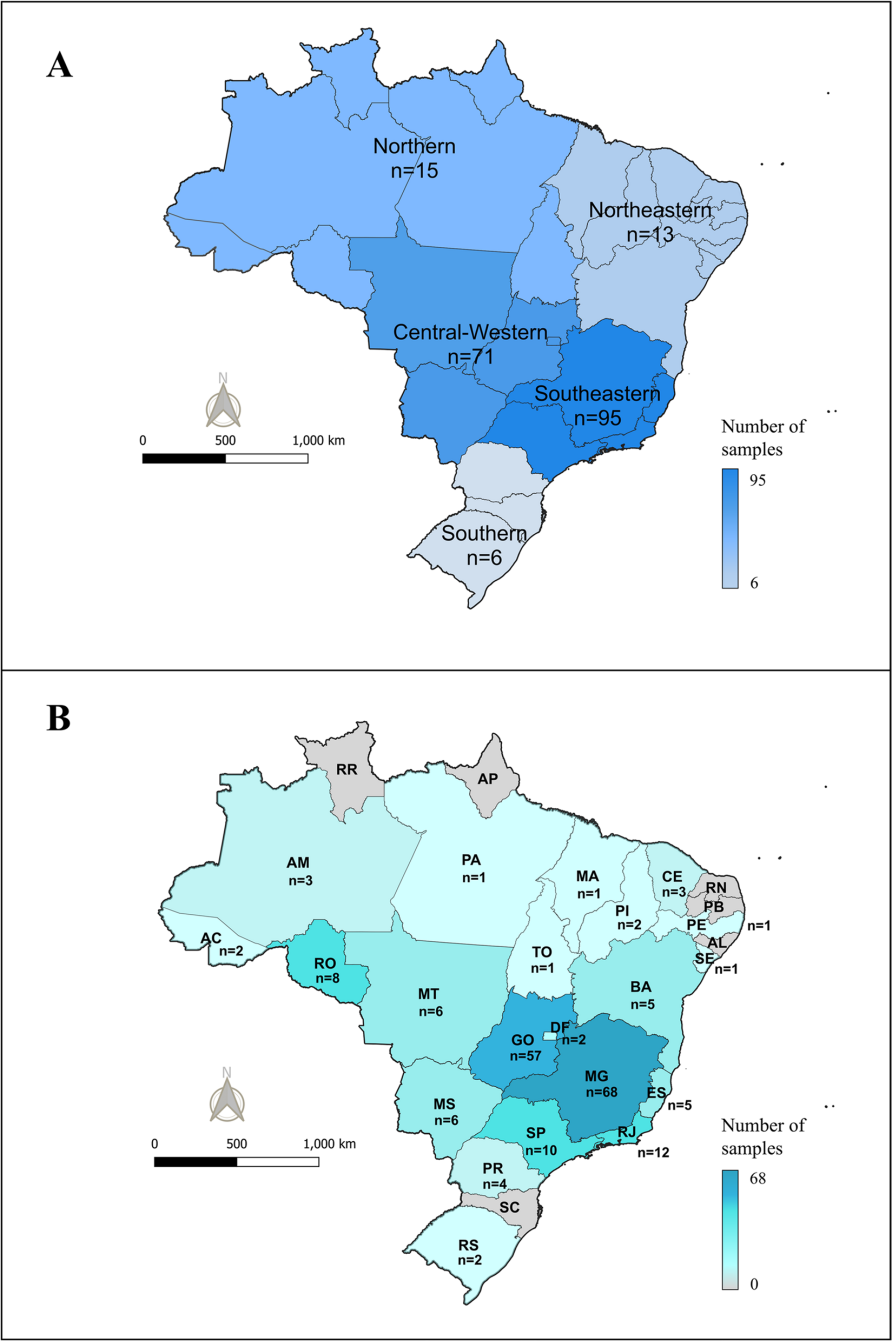
Origin of *Rhipicephalus microplus* populations used in the discriminating dose tests. **A** Samples grouped by region of Brazil. **B** Samples grouped by Brazilian federative units. *n* = number of samples per region or federative unit

The engorged females were collected from naturally infested cattle with no recent history of acaricide treatment from private farms in various regions of Brazil between December 2022 and July 2025 (Fig. 1). Most samples were provided by Parasitology Laboratory at Embrapa Dairy Cattle [27]. These samples were forwarded to the Laboratory of Biology, Ecology, and Tick Control (LABEC) at the Federal University of Goiás (UFG) for discriminating dose (DD) testing. Additional samples were either collected directly by LABEC researchers or obtained by collaborating researchers or farm workers and sent directly to the LABEC facility.

In the laboratory, engorged females from both reference strains and field samples were placed in Petri dishes for 14 days and maintained in a biochemical oxygen demand (BOD) incubator (SL-200/364, SOLAB) at 27 ± 1 °C and 80 ± 10% relative humidity (RH) for oviposition. The resulting egg masses were homogenized, and aliquots of 250 mg were weighed and stored in 5 mL plastic syringes, with the distal ends cut and sealed with absorbent cotton. The syringes were kept under the same controlled conditions to allow for larval hatching. Larvae aged between 14 and 28 days post-hatching were used in the tests.

### Fluralaner

In the laboratory tests, the commercial product Exzolt^®^ 5% (Batch: 200818-2, MSD Animal Health) was used. Initially, a stock solution was prepared at a concentration of 50 µg/mL, using 2% (v/v) dimethyl sulfoxide (DMSO) as the solvent. From this stock solution, two DDs (1.55 and 3.16 µg/mL) were prepared, as defined by Rodrigues et al. [14]. These DDs were determined as follows: (i) based on the 2 × LC99 value of the susceptible population (1.55 µg/mL) and (ii) based on the 2 × LC99 value using mortality data from 18 populations as a single sample (3.16 µg/mL). All solutions were prepared in 10 mL volumetric flasks and subsequently transferred to 1.5 mL microtubes for testing. For the field trial, the commercial product was applied according to the manufacturer’s recommendations (1 mL per 20 kg of body weight).

### Larval immersion test (LIT) with discriminating doses (DD)

The LIT was conducted according to the methodology proposed by Sabatini et al. [28], with modifications as described by Rodrigues et al. [14]. Approximately 500 unfed larvae of *R. microplus* were placed into microcentrifuge tubes containing each treatment solution, and the tubes were vigorously agitated for 3 min. Following this exposure period, the solution was filtered using a cloth filter, and approximately 100 larvae were transferred to 6 cm^2^ filter paper packets. These packets were then folded in half and sealed at the edges using binder clips.

The packets were incubated in a BOD incubator at 27 ± 1 °C and relative humidity of 80 ± 10%. After 24 ± 2 h, the number of live and dead larvae was counted. Larvae that were unable to move were considered dead. Each treatment was tested in five replicates, and the entire test was repeated on two separate days, resulting in a total of 10 packets per sample evaluated. For the reference populations (POA and GYN), tests were repeated on five different days, yielding a total of 25 packets per population. A negative control group was also established, in which larvae were exposed to the solvent alone (2% DMSO). Populations in which negative control mortality was less than 5% were included in the study.

### Field efficacy study

To compare the laboratory susceptibility and field efficacy results, in relation to the classification of the susceptibility profile, one farm located in the municipality of São José do Rio Pardo (São Paulo, Brazil) was selected. The tick population from this farm was designated as the SJRP population. This site was chosen due to its documented history of fluralaner use [13]. All clinical procedures throughout the experiment were conducted in accordance with Good Clinical Practice guidelines [29].

For the field trial, 20 Simmental cattle naturally infested with *R. microplus* were selected. Only animals with an average count of more than 20 ticks (measuring between 4.5 and 8.0 mm in length) on the left side of the body were included. The selected cattle had an average age of 48 months, had an average weight of 490 kg, and were in good nutritional condition with an appropriate body condition score. During the 90 days preceding the study, no antiparasitic treatments were administered, and the animals were maintained in paddocks with coast cross grass (*Cynodon dactylon*), with free access to water and mineral salt.

The selected animals were divided into two groups: T01, the control group (untreated animals), and T02, the treatment group (animals treated with Exzolt^®^ 5%). To form these groups, ticks measuring between 4.5 and 8.0 mm in length were counted on the left side of each animal for three consecutive days prior to the start of the experiment (days −3, −2, and −1). On the basis of these counts, the animals were ranked in descending order according to their mean tick count. Subsequently, the 20 selected animals were allocated alternately to each group, beginning with the animal with the highest average tick count, followed by the next highest, and so on, thereby ensuring the formation of two homogeneous groups with ten cattle each.

On day 0, animals belonging to the treatment group, T02, were weighed and individually treated according to their body weight, using the dosage recommended by the manufacturer (1 mL per 20 kg of body weight). Each animal received the treatment via a disposable syringe, with the full dose administered along the midline of the back, from the withers to the base of the tail.

Following treatment administration, tick counts were performed on days +3, +7, +14, +21, +28, +35, +42, +49, +56, and +63. Only ticks measuring between 4.5 and 8.0 mm in length were included in the counts [30, 31]. For the counts, the animals were physically restrained in a holding pen, and counts were performed only on the left side of the animals, by the same person, and between 8:00 and 10:00 in the morning, using a measuring board. Treatment efficacy was calculated using the formula described by Roulston and Wharton [32], which is also adopted by the Brazilian Ministry of Agriculture and Livestock [33], as outlined below:

$$Efficacy \left( \% \right) = \left[ {1 - \frac{Ta \times Cb}{{Tb \times Ca}}} \right] \times 100$$

where *Ta* is the average number of ticks on animals in the treated group (T02) after treatment; *Cb* is there average number of ticks on animals in the control group (T01) 3 days before treatment; *Tb* is the average number of ticks on animals in the treated group (T02) 3 days before treatment; and *Ca* is the average number of ticks on animals in the control group (T01) after the treatment date.

### Statistical analysis

For the discriminating dose (DD) assays, statistical comparisons of means were conducted of the same sample exposed to different treatments. Initially, the Shapiro–Wilk test was applied to assess the normality of data distribution for all samples. As the data did not follow a normal distribution, the non-parametric Kruskal–Wallis test was used, followed by the Dunn post hoc test, using R software (version 4.4.0, 2024). Populations exhibiting larval mortality rates greater than 95% were classified as susceptible to fluralaner, in accordance with the criteria used by Klafke et al. [21].

For the field trial, the average counts of partially engorged *R. microplus* females were log-transformed to meet the assumptions of normality, homogeneity of variances, residual independence, and randomness of observations. Tick counts were analyzed using Tukey’s test (*P* ≤ 0.05) through the general linear model (GLM) procedure in SAS software (version 9.4) [34]. The tick population was considered susceptible to treatment when therapeutic and persistent efficacy exceeded 90%, as defined by Salvador et al. [20].

## Results

### Larval immersion test with discriminating doses

Samples from populations across all regions of Brazil were tested, as follows: 15 samples from the North region (Acre [AC], Amazonas [AM], Pará [PA], Rondônia [RO], and Tocantins [TO]); 13 samples from the Northeast region (Bahia [BA], Ceará [CE], Maranhão [MA], Pernambuco [PE], Piauí [PI], and Sergipe [SE]); 71 samples from the Central-West region (Distrito Federal [DF], Goiás [GO], Mato Grosso [MT], and Mato Grosso do Sul [MS]); 95 samples from the Southeast region (Espírito Santo [ES], Minas Gerais [MG], Rio de Janeiro [RJ], and São Paulo [SP]); and six samples from the South region (Paraná [PR] and Rio Grande do Sul [RS]). Tests were carried out with samples from 21 federative units among the 27 existing in the country. In the Southeast and Central-West regions, the tests included at least two population from each state (Fig. 1; Table 1).

**Table 1:** Larval mortality rate (%) of 200 unfed *Rhipicephalus microplus* populations from five regions of Brazil, exposed to two discriminating doses (DD) of fluralaner under laboratory conditions (27 ± 1 °C and 80 ± 10% relative humidity)

Tick strains and regions	N	FU	Municipality	Larval mortality (%)		
				Control	DD: 1.55 µg/mL	DD: 3.16 µg/mL
Reference strains	1	RS	Porto Alegre (POA)	2.7 ± 2.4^a^	100 ± 0.0^b^	100 ± 0.0^b^
	2	GO	Goiânia (GYN)	0.1 ± 0.3^a^	100 ± 0.0^b^	100 ± 0.0^b^
North	3	AC	Rio Branco	4.9 ± 2.8^a^	100 ± 0.0^b^	100 ± 0.0^b^
	4	AC	Rio Branco	0.7 ± 1.4^a^	99.9 ± 0.5^b^	100 ± 0.0^b^
	5	AM	Autazes	4.4 ± 1.3^a^	100 ± 0.0^b^	100 ± 0.0^b^
	6	AM	Careiro da Várzea	4.1 ± 2.5^a^	100 ± 0.0^b^	100 ± 0.0^b^
	7	AM	Careiro da Várzea	4.9 ± 2.2^a^	99.8 ± 0.8^b^	100 ± 0.0^b^
	8	PA	Canaã dos Carajás	2.5 ± 2.1^a^	100 ± 0.0^b^	100 ± 0.0^b^
	9	RO	Alta Floresta D’Oeste	3.8 ± 3.1^a^	99.6 ± 1.3^b^	99.8 ± 0.8^b^
	10	RO	Espigão D’Oeste	1.0 ± 0.1^a^	100 ± 0.0^b^	100 ± 0.0^b^
	11	RO	Jaru	0.2 ± 0.3^a^	100 ± 0.0^b^	100 ± 0.0^b^
	12	RO	Ji-Paraná	4.6 ± 2.3^a^	100 ± 0.0^b^	100 ± 0.0^b^
	13	RO	Pimenta Bueno	0.8 ± 1.4^a^	100 ± 0.0^b^	100 ± 0.0^b^
	14	RO	Presidente Médici	1.5 ± 0.5^a^	100 ± 0.0^b^	100 ± 0.0^b^
	15	RO	Urupá	2.7 ± 1.1^a^	98.8 ± 0.4^b^	99.4 ± 0.6^b^
	16	RO	Rondolândia	3.1 ± 2.2^a^	97.4 ± 2.4^b^	100 ± 0.0^c^
	17	TO	Murilândia	4.9 ± 2.4^a^	100 ± 0.0^b^	100 ± 0.0^b^
Northeast	18	BA	Amargosa	2.5 ± 3.4^a^	99.1 ± 2.9^b^	100 ± 0.0^b^
	19	BA	Amargosa	4.4 ± 3.3^a^	100 ± 0.0^b^	100 ± 0.0^b^
	20	BA	Formosa do Rio Preto	4.3 ± 2.8^a^	100 ± 0.0^b^	100 ± 0.0^b^
	21	BA	Piripá	1.3 ± 1.5^a^	100 ± 0.0^b^	100 ± 0.0^b^
	22	BA	Vitória da Conquista	4.7 ± 3.1^a^	99.8 ± 0.5^b^	100 ± 0.0^b^
	23	CE	Aquiraz	2.4 ± 2.0^a^	100 ± 0.0^b^	100 ± 0.0^b^
	24	CE	Aquiraz	3.3 ± 4.2^a^	99.2 ± 1.4^b^	100 ± 0.0^b^
	25	CE	Moraguape	1.8 ± 2.0^a^	100 ± 0.0^b^	100 ± 0.0^b^
	26	MA	Caxias	2.2 ± 2.0^a^	100 ± 0.0^b^	100 ± 0.0^b^
	27	PE	Caruaru	4.5 ± 4.2^a^	99.0 ± 1.4^b^	100 ± 0.0^b^
	28	PI	Oeiras	2.3 ± 2.3^a^	100 ± 0.0^b^	100 ± 0.0^b^
	29	PI	Oeiras	1.5 ± 2.6^a^	100 ± 0.0^b^	100 ± 0.0^b^
	30	SE	Boquim	1.5 ± 1.4^a^	100 ± 0.0^b^	100 ± 0.0^b^
Central-West	31	DF	Brasília	3.6 ± 3.2^a^	100 ± 0.0^b^	100 ± 0.0^b^
	32	DF	Planaltina	4.4 ± 3.0^a^	100 ± 0.0^b^	100 ± 0.0^b^
	33	GO	Aragoiânia	3.1 ± 1.4^a^	99.7 ± 0.7^b^	100 ± 0.0^b^
	34	GO	Aragoiânia	3.4 ± 1.9^a^	100 ± 0.0^b^	100 ± 0.0^b^
	35	GO	Aragoiânia	2.1 ± 1.7^a^	100 ± 0.0^b^	100 ± 0.0^b^
	36	GO	Araçu	1.8 ± 1.6^a^	100 ± 0.0^b^	100 ± 0.0^b^
	37	GO	Araçu	0.3 ± 0.5^a^	100 ± 0.0^b^	100 ± 0.0^b^
	38	GO	Araçu	0.0 ± 0.0^a^	100 ± 0.0^b^	100 ± 0.0^b^
	39	GO	Araçu	0.0 ± 0.0^a^	100 ± 0.0^b^	100 ± 0.0^b^
	40	GO	Bela Vista de Goiás	2.3 ± 1.6^a^	100 ± 0.0^b^	100 ± 0.0^b^
	41	GO	Bela Vista de Goiás	4.1 ± 0.5^a^	99.7 ± 0.9^b^	100 ± 0.0^b^
	42	GO	Bela Vista de Goiás	2.2 ± 1.8^a^	100 ± 0.0^b^	100 ± 0.0^b^
	43	GO	Bela Vista de Goiás	4.4 ± 2.3^a^	98.8 ± 2.2^b^	100 ± 0.0^b^
	44	GO	Bela Vista de Goiás	4.8 ± 1.7^a^	99.1 ± 2.7^b^	100 ± 0.0^b^
	45	GO	Bela Vista de Goiás	1.7 ± 3.0^a^	100 ± 0.0^b^	100 ± 0.0^b^
	46	GO	Bela Vista de Goiás	1.0 ± 1.9^a^	100 ± 0.0^b^	100 ± 0.0^b^
	47	GO	Bela Vista de Goiás	0.7 ± 0.8^a^	100 ± 0.0^b^	100 ± 0.0^b^
	48	GO	Bela Vista de Goiás	4.0 ± 1.3^a^	100 ± 0.0^b^	100 ± 0.0^b^
	49	GO	Bela Vista de Goiás	1.0 ± 1.0^a^	100 ± 0.0^b^	100 ± 0.0^b^
	50	GO	Bela Vista de Goiás	0.6 ± 1.0^a^	100 ± 0.0^b^	100 ± 0.0^b^
	51	GO	Bela Vista de Goiás	0.0 ± 0.0^a^	97.1 ± 2.5^b^	100 ± 0.0^b^
	52	GO	Bela Vista de Goiás	0.3 ± 0.4^a^	98.4 ± 0.2^b^	97.7 ± 4.1^b^
	53	GO	Bela Vista de Goiás	3.4 ± 0.8^a^	100 ± 0.0^b^	99.5 ± 0.8^b^
	54	GO	Bela Vista de Goiás	2.7 ± 2.0^a^	100 ± 0.0^b^	100 ± 0.0^b^
	55	GO	Brazabrantes	1.7 ± 0.4^a^	100 ± 0.0^b^	100 ± 0.0^b^
	56	GO	Campestre de Goiás	4.6 ± 2.7^a^	100 ± 0.0^b^	100 ± 0.0^b^
	57	GO	Catalão	1.3 ± 2.9^a^	100 ± 0.0^b^	100 ± 0.0^b^
	58	GO	Catalão	1.9 ± 0.9^a^	100 ± 0.0^b^	100 ± 0.0^b^
	59	GO	Cristianópolis	1.1 ± 1.6^a^	100 ± 0.0^b^	100 ± 0.0^b^
	60	GO	Cumari	0.6 ± 1.0^a^	100 ± 0.0^b^	100 ± 0.0^b^
	61	GO	Cumari	3.8 ± 1.3^a^	100 ± 0.0^b^	100 ± 0.0^b^
	62	GO	Goiânia	2.4 ± 4.1^a^	100 ± 0.0^b^	100 ± 0.0^b^
	63	GO	Goiânia	2.3 ± 1.4^a^	100 ± 0.0^b^	100 ± 0.0^b^
	64	GO	Goiânia	1.8 ± 0.8^a^	100 ± 0.0^b^	100 ± 0.0^b^
	65	GO	Goianápolis	0.0 ± 0.0^a^	99.1 ± 1.6^b^	100 ± 0.0^b^
	66	GO	Goianápolis	1.6 ± 2.1	100 ± 0.0^b^	100 ± 0.0^b^
	67	GO	Hidrolândia	0.3 ± 1.1^a^	100 ± 0.0^b^	100 ± 0.0^b^
	68	GO	Inhumas	4.0 ± 3.1^a^	100 ± 0.0^b^	100 ± 0.0^b^
	69	GO	Inhumas	4.1 ± 1.8^a^	100 ± 0.0^b^	100 ± 0.0^b^
	70	GO	Itaberaí	1.8 ± 0.8^a^	100 ± 0.0^b^	100 ± 0.0^b^
	71	GO	Itumbiara	4.1 ± 2.8^a^	99.2 ± 1.4^b^	98.9 ± 1.3^b^
	72	GO	Itumbiara	1.5 ± 1.4^a^	97.3 ± 1.1^b^	100 ± 0.0^b^
	73	GO	Moiporá	3.5 ± 1.3^a^	100 ± 0.0^b^	100 ± 0.0^b^
	74	GO	Montes Claros de Goiás	4.2 ± 2.7^a^	100 ± 0.0^b^	100 ± 0.0^b^
	75	GO	Quirinópolis	1.3 ± 1.3^a^	100 ± 0.0^b^	100 ± 0.0^b^
	76	GO	Rio Verde	4.1 ± 1.8 ±	100 ± 0.0^b^	100 ± 0.0^b^
	77	GO	Rio Verde	3.6 ± 2.4^a^	100 ± 0.0^b^	100 ± 0.0^b^
	78	GO	Rio Verde	0.0 ± 0.0^a^	100 ± 0.0^b^	100 ± 0.0^b^
	79	GO	Santa Fé de Goiás	0.0 ± 0.0^a^	100 ± 0.0^b^	100 ± 0.0^b^
	80	GO	Santa Rosa de Goiás	3.3 ± 2.9^a^	100 ± 0.0^b^	100 ± 0.0^b^
	81	GO	São João da Paraúna	1.9 ± 1.9^a^	100 ± 0.0^b^	100 ± 0.0^b^
	82	GO	São Miguel do Passa Quatro	0.0 ± 0.0^a^	100 ± 0.0^b^	100 ± 0.0^b^
	83	GO	São Miguel do Passa Quatro	0.0 ± 0.0^a^	99.1 ± 1.5^b^	98.7 ± 2.3^b^
	84	GO	São Miguel do Passa Quatro	1.2 ± 0.8^a^	100 ± 0.0^b^	100 ± 0.0^b^
	85	GO	São Miguel do Passa Quatro	3.5 ± 2.7^a^	100 ± 0.0^b^	100 ± 0.0^b^
	86	GO	São Miguel do Passa Quatro	0.8 ± 0.5^a^	100 ± 0.0^b^	100 ± 0.0^b^
	87	GO	Silvânia	1.4 ± 1.2^a^	100 ± 0.0^b^	100 ± 0.0^b^
	88	GO	Urupá	1.6 ± 0.6^a^	98.9 ± 1.2^b^	99.3 ± 1.3^b^
	89	MS	Camapuã	4.1 ± 3.0^a^	100 ± 0.0^b^	100 ± 0.0^b^
	90	MS	Campo Grande	3.9 ± 2.6^a^	100 ± 0.0^b^	100 ± 0.0^b^
	91	MS	Cassilândia	3.5 ± 2.9^a^	100 ± 0.0^b^	100 ± 0.0^b^
	92	MS	Cassilândia	4.6 ± 1.4^a^	100 ± 0.0^b^	100 ± 0.0^b^
	93	MS	Corguinho	4.4 ± 3.4^a^	100 ± 0.0^b^	100 ± 0.0^b^
	94	MS	Miranda	3.5 ± 2.6^a^	100 ± 0.0^b^	100 ± 0.0^b^
	95	MT	Cuiabá	1.5 ± 2.0^a^	100 ± 0.0^b^	98.8 ± 2.6^b^
	96	MT	Itanhangá	2.9 ± 2.5^a^	100 ± 0.0^b^	100 ± 0.0^b^
	97	MT	Alta Floresta	3.5 ± 3.5^a^	100 ± 0.0^b^	100 ± 0.0^b^
	98	MT	Alta Floresta	3.3 ± 3.3^a^	100 ± 0.0^b^	100 ± 0.0^b^
	99	MT	Vale Azul	0.8 ± 1.2^a^	99.8 ± 0.6^b^	100 ± 0.0^b^
	100	MT	Vale Azul	1.0 ± 1.3^a^	100 ± 0.0^b^	100 ± 0.0^b^
Southeast	101	ES	Alegre	2.9 ± 3.3^a^	99.6 ± 1.3^b^	100 ± 0.0^b^
	102	ES	Alegre	3.7 ± 3.0^a^	100 ± 0.0^b^	100 ± 0.0^b^
	103	ES	Apiacá	0.0 ± 0.0^a^	98.9 ± 1.2^b^	99.3 ± 1.2^b^
	104	ES	Santa Teresa	3.6 ± 1.5^a^	100 ± 0.0^b^	100 ± 0.0^b^
	105	ES	Muniz Freire	2.3 ± 2.0^a^	100 ± 0.0^b^	100 ± 0.0^b^
	106	MG	Alto Rio Doce	1.2 ± 2.1^a^	100 ± 0.0^b^	100 ± 0.0^b^
	107	MG	Andrelândia	3.3 ± 1.4^a^	100 ± 0.0^b^	100 ± 0.0^b^
	108	MG	Arceburgo	2.7 ± 2.6^a^	100 ± 0.0^b^	100 ± 0.0^b^
	109	MG	Barbacena	1.1 ± 1.2^a^	100 ± 0.0^b^	100 ± 0.0^b^
	110	MG	Belo Vale	3.0 ± 2.8^a^	100 ± 0.0^b^	100 ± 0.0^b^
	111	MG	Bicas	4.5 ± 1.0^a^	100 ± 0.0^b^	100 ± 0.0^b^
	112	MG	Bom Sucesso	0.0 ± 0.0^a^	100 ± 0.0^b^	100 ± 0.0^b^
	113	MG	Bonfinópolis de Minas	1.6 ± 1.7^a^	100 ± 0.0^b^	100 ± 0.0^b^
	114	MG	Braúnas	0.0 ± 0.0^a^	100 ± 0.0^b^	100 ± 0.0^b^
	115	MG	Caldas	2.6 ± 1.5^a^	100 ± 0.0^b^	100 ± 0.0^b^
	116	MG	Campina Verde	4.8 ± 3.3^a^	100 ± 0.0^b^	100 ± 0.0^b^
	117	MG	Campo Florido	4.3 ± 2.5^a^	99.8 ± 0.6^b^	100 ± 0.0^b^
	118	MG	Caranaíba	1.9 ± 2.0^a^	100 ± 0.0^b^	100 ± 0.0^b^
	119	MG	Carvalhos	2.0 ± 2.0^a^	100 ± 0.0^b^	100 ± 0.0^b^
	120	MG	Cataguases	0.6 ± 0.8^a^	99.6 ± 0.7^b^	99.5 ± 0.8^b^
	121	MG	Catas Altas	1.3 ± 1.2^a^	100 ± 0.0^b^	100 ± 0.0^b^
	122	MG	Conceição da Barra de Minas	4.9 ± 3.2^a^	100 ± 0.0^b^	100 ± 0.0^b^
	123	MG	Contagem	0.6 ± 0.7^a^	100 ± 0.0^b^	100 ± 0.0^b^
	124	MG	Coronel Pacheco	4.9 ± 3.2^a^	98.8 ± 2.1^b^	100 ± 0.0^b^
	125	MG	Divinópolis	2.9 ± 1.5^a^	100 ± 0.0^b^	100 ± 0.0^b^
	126	MG	Dores do Turvo	1.7 ± 1.8^a^	100 ± 0.0^b^	100 ± 0.0^b^
	127	MG	Entre Rios de Minas	3.7 ± 3.1^a^	100 ± 0.0^b^	100 ± 0.0^b^
	128	MG	Guarará	1.2 ± 2.1^a^	100 ± 0.0^b^	100 ± 0.0^b^
	129	MG	Guarará	1.1 ± 1.9^a^	100 ± 0.0^b^	100 ± 0.0^b^
	130	MG	Guarará	2.4 ± 4.1^a^	100 ± 0.0^b^	100 ± 0.0^b^
	131	MG	Guarará	1.4 ± 1.3^a^	100 ± 0.0^b^	100 ± 0.0^b^
	132	MG	Guarará	0.6 ± 1.0^a^	100 ± 0.0^b^	100 ± 0.0^b^
	133	MG	Gurinhatã	4.8 ± 3.1^a^	100 ± 0.0^b^	99.1 ± 2.3^b^
	134	MG	Iraí de Minas	2.9 ± 2.0^a^	100 ± 0.0^b^	100 ± 0.0^b^
	135	MG	Ingaí	3.1 ± 3.6^a^	100 ± 0.0^b^	100 ± 0.0^b^
	136	MG	Itamarandiba	0.9 ± 0.7^a^	100 ± 0.0^b^	100 ± 0.0^b^
	137	MG	Itapagipe	4.4 ± 6.4^a^	100 ± 0.0^b^	100 ± 0.0^b^
	138	MG	Ituiutaba	2.2 ± 1.0^a^	100 ± 0.0^b^	100 ± 0.0^b^
	139	MG	Janúba	3.1 ± 3.2^a^	100 ± 0.0^b^	100 ± 0.0^b^
	140	MG	Juiz de Fora	0.3 ± 0.6^a^	98.9 ± 1.0^b^	99.0 ± 1.7^b^
	141	MG	Juiz de Fora	0.7 ± 1.3^a^	99.5 ± 0.9^b^	100 ± 0.0^b^
	142	MG	Juiz de Fora	4.0 ± 1.4^a^	98.9 ± 1.9^b^	99.5 ± 0.9^b^
	143	MG	Juiz de Fora	1.1 ± 0.5^a^	100 ± 0.0^b^	100 ± 0.0^b^
	144	MG	Juiz de Fora	3.5 ± 1.4^a^	100 ± 0.0^b^	100 ± 0.0^b^
	145	MG	Juiz de Fora	0.8 ± 0.4^a^	100 ± 0.0^b^	100 ± 0.0^b^
	146	MG	Lagamar	2.8 ± 1.1^a^	100 ± 0.0^b^	100 ± 0.0^b^
	147	MG	Lima Duarte	1.3 ± 1.6^a^	100 ± 0.0^b^	100 ± 0.0^b^
	148	MG	Lima Duarte	2.4 ± 2.1^a^	100 ± 0.0^b^	100 ± 0.0^b^
	149	MG	Lima Duarte	3.6 ± 1.1^a^	100 ± 0.0^b^	100 ± 0.0^b^
	150	MG	Lima Duarte	2.0 ± 2.5^a^	100 ± 0.0^b^	100 ± 0.0^b^
	151	MG	Nova Lima	1.7 ± 0.6^a^	100 ± 0.0^b^	100 ± 0.0^b^
	152	MG	Oliveira	1.9 ± 1.2^a^	100 ± 0.0^b^	100 ± 0.0^b^
	153	MG	Patos de Minas	1.7 ± 1.9^a^	100 ± 0.0^b^	100 ± 0.0^b^
	154	MG	Pedro Leopoldo	3.9 ± 1.8^a^	100 ± 0.0^b^	100 ± 0.0^b^
	155	MG	Pedro Teixeira	0.0 ± 0.0^a^	100 ± 0.0^b^	100 ± 0.0^b^
	156	MG	Piau	0.0 ± 0.0^a^	98.2 ± 2.1^b^	100 ± 0.0^b^
	157	MG	Pitangui	2.2 ± 1.8^a^	100 ± 0.0^b^	100 ± 0.0^b^
	158	MG	Presidente Olegário	5.3 ± 9.1^a^	100 ± 0.0^b^	100 ± 0.0^b^
	159	MG	Presidente Olegário	4.3 ± 0.7^a^	100 ± 0.0^b^	100 ± 0.0^b^
	160	MG	Ressaquinha	3.1 ± 2.9^a^	98.8 ± 2.1^b^	100 ± 0.0^b^
	161	MG	Rubim	2.4 ± 2.2^a^	100 ± 0.0^b^	100 ± 0.0^b^
	162	MG	Sabará	3.1 ± 2.7^a^	100 ± 0.0^b^	100 ± 0.0^b^
	163	MG	Sabará	4.5 ± 0.4^a^	100 ± 0.0^b^	100 ± 0.0^b^
	164	MG	Santa Juliana	1.0 ± 1.2^a^	100 ± 0.0^b^	100 ± 0.0^b^
	165	MG	Serra Azul de Minas	0.6 ± 0.5^a^	100 ± 0.0^b^	100 ± 0.0^b^
	166	MG	São João Del Rei	1.0 ± 1.1^a^	100 ± 0.0^b^	100 ± 0.0^b^
	167	MG	São João Nepomuceno	0.6 ± 0.6^a^	100 ± 0.0^b^	100 ± 0.0^b^
	168	MG	Turvolândia	2.9 ± 2.8^a^	98.1 ± 3.3^b^	100 ± 0.0^b^
	169	MG	Uberlândia	2.6 ± 3.5^a^	100 ± 0.0^b^	100 ± 0.0^b^
	170	MG	Uberlândia	0.2 ± 0.5^a^	100 ± 0.0^b^	100 ± 0.0^b^
	171	MG	Unaí	2.9 ± 2.3^a^	100 ± 0.0^b^	100 ± 0.0^b^
	172	MG	Unaí	4.3 ± 2.1^a^	100 ± 0.0^b^	100 ± 0.0^b^
	173	MG	Vazante	0.0 ± 0.0^a^	100 ± 0.0^b^	100 ± 0.0^b^
	174	RJ	Araruama	3.1 ± 3.1^a^	100 ± 0.0^b^	100 ± 0.0^b^
	175	RJ	Bom Jardim	2.3 ± 1.1^a^	99.7 ± 0.4^b^	100 ± 0.0^b^
	176	RJ	Bom Jesus do Itabapoana	4.1 ± 3.1^a^	100 ± 0.0^b^	100 ± 0.0^b^
	177	RJ	Cachoeira de Macacu	1.9 ± 2.4^a^	100 ± 0.0^b^	100 ± 0.0^b^
	178	RJ	Cachoeira de Macacu	2.9 ± 2.4^a^	100 ± 0.0^b^	100 ± 0.0^b^
	179	RJ	Cachoeira de Macacu	4.6 ± 1.1^a^	100 ± 0.0^b^	100 ± 0.0^b^
	180	RJ	Rio de Janeiro	4.3 ± 4.2^a^	100 ± 0.0^b^	100 ± 0.0^b^
	181	RJ	Santa Maria Madalena	0.0 ± 0.0^a^	100 ± 0.0^b^	100 ± 0.0^b^
	182	RJ	Valença	4.5 ± 2.6^a^	100 ± 0.0^b^	100 ± 0.0^b^
	183	RJ	Valença	0.0 ± 0.0^a^	97.3 ± 1.3^b^	99.7 ± 0.5^b^
	184	RJ	Valença	1.8 ± 0.3^a^	100 ± 0.0^b^	100 ± 0.0^b^
	185	RJ	Valença	± 0.0^a^	96.9 ± 2.9^b^	100 ± 0.0^b^
	186	SP	General Salgado	1.0 ± 1.9^a^	100 ± 0.0^b^	98.9 ± 1.9^b^
	187	SP	Guaimbê	0.0 ± 0.0^a^	100 ± 0.0^b^	98.9 ± 0.4^b^
	189	SP	José Bonifácio	1.2 ± 2.1^a^	99.3 ± 1.3^b^	100 ± 0.0^b^
	190	SP	Pereira Barreto	1.1 ± 2.1^a^	100 ± 0.0^b^	100 ± 0.0^b^
	191	SP	São Carlos	2.3 ± 1.9^a^	100 ± 0.0^b^	100 ± 0.0^b^
	192	SP	São Carlos	4.7 ± 3.2^a^	100 ± 0.0^b^	100 ± 0.0^b^
	193	SP	São Carlos	1.7 ± 1.8^a^	98.7 ± 3.2^b^	100 ± 0.0^b^
	194	SP	São Carlos	2.7 ± 2.1^a^	100 ± 0.0^b^	100 ± 0.0^b^
	*195*	*SP*	*São José do Rio Pardo (SJRP)*	*4.2* ± *5.3*^*a*^	*98.4* ± *3.8*^*b*^	*100* ± *0.0*^*b*^
South	196	PR	Cidade Gaúcha	3.5 ± 3.3^a^	100 ± 0.0^b^	100 ± 0.0^b^
	197	PR	Cidade Gaúcha	2.2 ± 2.7^a^	100 ± 0.0^b^	100 ± 0.0^b^
	198	PR	Maringá	1.5 ± 0.4^a^	99.1 ± 1.6^b^	100 ± 0.0^b^
	199	PR	Maringá	0.9 ± 1.6^a^	99.1 ± 1.5^b^	99.1 ± 1.6^b^
	200	RS	São Sepé	1.8 ± 2.1^a^	100 ± 0.0^b^	100 ± 0.0^b^

Means followed by different letters (a and b) in the same row differ significantly (*P* < 0.05) at the 5% significance level (Kruskal–Wallis). POA is an acaricide-susceptible strain, GYN is an acaricide-resistant strain (synthetic pyrethroids, formamidines, organophosphates, and phenylpyrazoles). SJRP (indicated in italics) is a population selected for field study. Control refers to the control group, which was exposed to 2% dimethyl sulfoxide (DMSO) alone. *DD* discriminating dose, *FU* Brazilian federative units, *AC* Acre, *AM* Amazonas, *BA* Bahia, *CE* Ceará, *DF* Distrito Federal, *ES* Espírito Santo, *GO* Goiás, *MA* Maranhão, *MG* Minas Gerais, *MS* Mato Grosso do Sul, *MT* Mato Grosso, *PA* Pará, *PE* Pernambuco, *PI* Piauí, *PR* Paraná, *RJ* Rio de Janeiro, *RO* Rondônia, *RS* Rio Grande do Sul, *SE* Sergipe, *SP* São Paulo, *TO* Tocantins

Larval mortality rates in the negative control group across all regions ranged from 0% to 4.9%, indicating that the populations were suitable for testing. Significant differences (*P* < 0.01) were observed in mortality values with two DDs, when compared with the values observed in the negative control, across all populations (Table 1).

In the tests with the two reference strains (POA and GYN), 100% mortality was observed for both DDs tested (Table 1). For the SJRP population, mortality was 98.4% and 100% for the DDs of 1.55 and 3.16 µg/mL, respectively. In the remaining populations, the DD at a concentration of 1.55 µg/mL resulted in 100% mortality in 160 populations (80%), whereas the DD at a concentration of 3.16 µg/mL showed more consistent results, with 100% mortality in 182 populations (91%). All populations tested, regardless of the DD, exhibited mortality rates exceeding 95% (Table 1).

### Field efficacy study

In the field trial, cattle treated with fluralaner (T02) exhibited significantly lower mean tick counts compared with the control group (T01) starting on day +3 (*P* < 0.0001) and continuing through day +56 (*P* < 0.0033) (Table 2). From days +7 to +42, no ticks measuring between 4.5 and 8.0 mm were observed on treated animals. Treatment efficacy on day +3 was 87.8%, reaching 100% by day +7 and maintaining this level through day +42. On day +49, efficacy was 98.9%, declining to 75.1% on day +56 and 14.0% on day +63 (Table 2). Therapeutic efficacy, assessed from days +7 to +21, and persistent efficacy, from days +28 to +42, were both 100% (Table 2). These findings confirm that the SJRP population is phenotypically susceptible to fluralaner.

**Table 2:** Arithmetic means of *Rhipicephalus microplus* female counts (measuring between 4.5 and 8.0 mm in length) on cattle and the efficacy of fluralaner under field conditions on a private farm located in São José do Rio Pardo, São Paulo, Brazil (SJRP tick population)

Days	Experimental groups/Mean number of *R. microplus* females (4.5–8 mm)		*P*-value	CV	Efficacy (%)
	T01: Control	T02: Fluralaner^**^			T02
0^*^	28.87^a^	28.07^a^	0.9884	6.32	—
3	31.20^a^	3.70^b^	< 0.0001	37.3	87.8
7	33.10^a^	0.00^b^	< 0.0001	28.01	100.0
14	34.80^a^	0.00^b^	< 0.0001	28.98	100.0
21	22.80^a^	0.00^b^	< 0.0001	22.28	100.0
28	20.90^a^	0.00^b^	< 0.0001	20.35	100.0
35	21.00^a^	0.00^b^	< 0.0001	17.61	100.0
42	19.10^a^	0.00^b^	< 0.0001	19.5	100.0
49	19.20^a^	0.20^b^	< 0.0001	60.28	98.9
56	19.00^a^	4.60^b^	0.0033	68.95	75.1
63	17.70^a^	14.80^a^	0.1141	58.01	14.0
		Therapeutic efficacy (7–21 days)			100.0
		Persistent efficacy (28–42 days)			100.0

*CV* Coefficient of variation

Means followed by different letters (a and b) in the same row indicate significant differences at the 5% level

^*^Mean of the tick counts between days −3, −2, and −1, prior to treatment

^**^Exzolt 5%^®^: 2.5 mg/kg (MSD Animal Health)

## Discussion

Laboratory tests have been used to assess the susceptibility of *R. microplus* populations to acaricides; however, recent studies have demonstrated divergence between some laboratory and field results [19, 20, 23–25], indicating the need for new laboratory test proposals [14]. In this study, we used two new DDs proposals with fluralaner [14] to evaluate susceptibility of *R. microplus* populations samples from all regions of Brazil, totaling 200 populations. In the laboratory, the results indicated that all populations tested were susceptible to this isoxazoline. Of the 200 populations, we selected one population with a documented history of fluralaner exposure (SJRP) and performed a field trial that confirmed susceptibility this population.

In assays conducted with both DDs, all populations exhibited mortality rates above 95%. However, the DD of 3.16 µg/mL achieved 100% mortality in 91% of the tested samples and was therefore considered the most appropriate for monitoring the susceptibility profile of *R. microplus* populations to fluralaner. This is because fluralaner was introduced to the market relatively recently (2022); therefore, it is naturally expected that most populations will exhibit 100% mortality in laboratory tests using DDs. These results suggest that defining a DD based on 2 × the mean LC99 of multiple populations, considering the natural variability that may occur among individuals of the same species [14], may be more appropriate than relying on 2 × the LC99 of a single susceptible strain, as proposed in earlier guidelines [1].

Brazil is divided into five regions and 27 federative units, but most studies on tick susceptibility to acaricides have been limited to a few regions, with nationwide analyses remaining scarce [21, 22, 35–39]. In some federative units such as Amazonas, Amapá, and Acre (samples from Acre and Amazonas were evaluated in the present study), no studies on tick susceptibility to acaricides had been conducted previously, highlighting the need for broader investigations [2]. To the best of our knowledge, this is the first study to evaluate tick populations from all five Brazilian regions (North, Northeast, Central-West, Southeast, and South) and from 21 federative units, including the first assessments in *R. microplus* populations from the states of Amazonas and Acre. Although the number of samples in the current investigation was limited in some regions/federative units, this represents an initial effort to assess susceptibility to fluralaner with broad geographical coverage. These data are essential for the ongoing susceptibility monitoring of *R. microplus* populations to this isoxazoline, providing a baseline of susceptibility close to the time of fluralaner’s market introduction in Brazil [13, 14]. They may serve as a foundation for continuous spatial and temporal monitoring of susceptibility of *R. microplus* populations to acaricides. It should be noted that the high number of samples being processed within a relatively short time frame (2 years) was possible because of the adoption of discriminating doses (DDs).

As mentioned, the results of the laboratory tests indicated that none of the evaluated populations were resistant to fluralaner. These results are consistent with previously published data on the susceptibility of ticks to fluralaner, obtained from studies conducted in different countries and tick species parasitizing cattle [14, 19, 40]. This outcome was expected, given that the molecule was only introduced to the Brazilian market in the second half of 2022 [13], and most of these populations had little or no prior exposure to fluralaner. Tick resistance to acaricides is an evolutionary response that arises after continuous exposure to a given compound, which eliminates susceptible individuals. This allows resistant individuals to reproduce, increasing the frequency of alleles associated with the resistance phenotype in each subsequent generation exposed to the acaricide, until the resistant genotype becomes predominant in the population [1, 16, 17].

Although susceptibility to fluralaner was anticipated among *R. microplus* populations, it is important to note that recent studies have reported cross-resistance between synthetic pyrethroids (SPs) and fluralaner in *Musca domestica* [41, 42]. This cross-resistance has been attributed to detoxification mediated by cytochrome P450 (CYP) enzymes and reduced cuticular penetration rates [42]. Among the 200 populations studied, only the GYN strain had been previously characterized as resistant to SPs [25]. However, resistance to this class of compounds has been widely documented in *R. microplus* populations from various regions of Brazil [21, 25, 37, 38, 43–48]. In studies involving over 100 *R. microplus* populations, resistance prevalence to SPs exceeded 97% [21, 22, 37, 44]. Therefore, it is likely that more than 95% of the 200 populations assessed in the present study are resistant to SPs. Nonetheless, none of them were resistant to fluralaner, suggesting the absence of cross-resistance between fluralaner and SPs in *R. microplus*. However, specific studies are needed to confirm this hypothesis.

A field test was conducted on the farm where the SJRP population originates, which has a history of exposure to this isoxazoline [13, 49], to compare the laboratory and field results. In this test, the therapeutic efficacy (+7 to +21 days) and persistent efficacy (+28 to +42 days) were both 100%, which were similar to those observed in other field studies with fluralaner in different regions of Brazil [13, 14]. These data confirm the laboratory results, classifying the SJRP population as susceptible. Rodrigues et al. [14] tested three *R. microplus* populations and also observed consistency between laboratory and field results using these two discriminating doses (DDs) of fluralaner. These findings are interesting, revealing that the proposed DDs have initially provided results that are consistent with field conditions. However, it is important to note that the current scenario may favor these results, given that the molecule was recently launched in the market, and thus, *R. microplus* populations show high susceptibility to this isoxazoline in field trials [13, 49–52]. Therefore, ongoing monitoring is essential to assess this methodology over time, especially when the first populations exhibiting some level of resistance begin to emerge, allowing the evaluation of the methodology under more challenging conditions and determining whether the consistency between laboratory and field results will persist [19, 20].

Although field results should be considered conclusive [10, 14, 19, 20, 23, 24], it is important to highlight that such studies are labor-intensive and expensive, making it difficult to conduct them on multiple populations with different acaricides. However, while laboratory tests are easier to conduct and allow for the evaluation of many populations and different acaricides [2, 16], it is worth noting that recent studies have shown inconsistencies between laboratory and field results [14, 19, 20, 23, 24]. Therefore, continuous investigation is necessary, comparing results from laboratory bioassays and field trials to confirm the susceptibility profile of a given population. This will enable the refinement, validation, and improvement of laboratory testing methodologies, providing more reliable and accurate results [10, 14, 20, 53]. The results presented here may provide a useful tool for monitoring the susceptibility profile of *R. microplus* populations to fluralaner. However, long-term studies are needed, including comparative analyses of laboratory and field data to validate the methods. Based on continuous monitoring, if divergence arises between laboratory and field results, field data should be considered the definitive reference for determining the susceptibility profile of a given population [10, 14, 20, 24].

## Conclusions

The phenotypic characterization results using discriminating doses (DDs) indicated that all 200 populations, originating from different regions of Brazil, are susceptible to fluralaner. The DD at a concentration of 3.16 µg/mL was shown to be the most appropriate for monitoring the susceptibility of *R. microplus* populations to fluralaner. The field test with the SJRP strain confirmed the results of the laboratory test, allowing us to conclude that this population is susceptible to fluralaner, suggesting a complementary role of two types of assessments (laboratory and field). These results can serve as a basis for continuous spatial and temporal monitoring of the susceptibility of *R. microplus* populations to fluralaner. Continued studies comparing DD results with field data are necessary to strengthen the evidence validating these laboratory test proposals. We reinforce that for resistance studies, especially for the first report of resistance for a compound/class acaricide, it is necessary carry out studies under both laboratory and field conditions, and field results should be considered as the final proof in diagnosing the susceptibility of *R. microplus* populations to acaricides. In addition, we emphasize that the DDs can only be definitively validated with the emergence of the first reports of resistance as well as convergent results from both laboratory and field trials in both susceptible and resistant populations.

## Data Availability

All data are included as tables and figures in the article.
